# Enhanced Osteogenic Differentiation of Periodontal Ligament Stem Cells Using a Graphene Oxide-Coated Poly(ε-caprolactone) Scaffold

**DOI:** 10.3390/polym13050797

**Published:** 2021-03-05

**Authors:** Jiyong Park, Sangbae Park, Jae Eun Kim, Kyoung-Je Jang, Hoon Seonwoo, Jong Hoon Chung

**Affiliations:** 1 Department of Biosystems Engineering, College of Agriculture and Life Sciences, Seoul National University, Seoul 08826, Korea; authorita@snu.ac.kr (J.P.); je6740@snu.ac.kr (J.E.K.); 2 Department of Biosystems & Biomaterials Science and Engineering, Seoul National University, Seoul 08826, Korea; sb92park@snu.ac.kr; 3 Division of Agro-System Engineering, College of Agriculture and Life Science, Gyeongsang National University, Jinju 52828, Korea; kj_jang@gnu.ac.kr; 4 Institute of Agriculture & Life Science, Gyeongsang National University, Jinju 52828, Korea; 5 Department of Industrial Machinery Engineering, College of Life Sciences and Natural Resources, Sunchon National University, Suncheon 57922, Korea; 6 Interdisciplinary Program in IT-Bio Convergence System, Sunchon National University, Suncheon 57922, Korea; 7 Research Institute of Agriculture and Life Sciences, Seoul National University, Seoul 08826, Korea; 8 BK21 Global Smart Farm Educational Research Center, Seoul National University, Seoul 08826, Korea

**Keywords:** 3D printing, poly(ε-caprolactone), graphene oxide, periodontal ligament stem cells, osteogenic differentiation

## Abstract

Periodontal diseases occur through bacterial infection in the oral cavity, which can cause alveolar bone loss. Several efforts have been made to reconstruct alveolar bone, such as grafting bone substitutes and 3D-printed scaffolds. Poly(ε-caprolactone) (PCL) is biocompatible and biodegradable, thus demonstrating its potential as a biomaterial substitute; however, it is difficult for cells to adhere to PCL because of its strong hydrophobicity. Therefore, its use as a biomaterial has limitations. In this study, we used graphene oxide (GO) as a coating material to promote the osteogenic differentiation ability of PCL scaffolds. First, 3D-printed PCL scaffolds were fabricated, and the oxygen plasma treatment and coating conditions were established according to the concentration of GO. The physical and chemical properties of the prepared scaffolds were evaluated through water contact angle analysis, Raman spectroscopy, and image analysis. In addition, the adhesion and proliferation of periodontal ligament stem cells (PDLSCs) on the GO scaffolds were assessed via the water-soluble tetrazolium salt-1 (WST-1) assay, and the osteogenic differentiation ability was evaluated through alizarin red S staining. The results confirmed that the cell proliferation and osteogenic differentiation of the PDLSCs were enhanced in the scaffolds coated with oxygen plasma and GO. In conclusion, the plasma-treated GO-coating method that we developed can be used to promote the cell proliferation and osteogenic differentiation of the scaffolds.

## 1. Introduction

Because of population aging, the number of elderly patients suffering from periodontal disease is increasing. Periodontal disease is broken down into four stages and the first stage is called gingivitis. Therefore, research on gingivitis is important to treat periodontal diseases. When periodontal diseases get worse, alveolar bone defects occur and various bone graft materials need to be transplanted for treatment [1]. However, this transplantation method can cause various side effects depending on the materials used [2]. As apical periodontitis is associated with periodontal pathology, which can cause bone defects, a regenerative approach must be considered [3]. The regeneration of periodontal tissue and the development of substitute materials are important topics in the field of tissue engineering [4]. Periodontal tissue is a complex tissue composed of cementum, periodontal ligament, alveolar bone, and gingiva. As these four tissues must be organically restored to regenerate periodontal tissue, and research on regenerating this tissue using stem cells is actively underway [5].

With the development of 3D printing technology, treatments utilizing 3D printing technology in the field of tissue engineering are being evaluated [6], and research on the development of biomaterials continues. Various bone substitute, such as ceramics and metals [7], are being studied [8,9]. Even though metals and ceramics have good physical properties for bone substitute materials, surgery is required for the removal of implants after the fracture has healed. In addition, it is difficult to attach cells on the surface of metals and ceramics. Therefore, there is a limitation to regenerate cells. Polymers are available in a wide variety of compositions, properties, and forms and can be readily fabricated into complex shapes and structures, which make them advantageous for use as biomaterials [10]. In addition, the polymer degrades naturally from the body because of the biodegradable properties; thus, surgical removal is not required [11]. Recently, composite materials including biodegradable matrix and bioactive fillers have been studied as cutting-edge technologies in the field of bone reconstruction [12].

Among the various biomaterials, polycaprolactone (PCL) is widely used in 3D printing for the fabrication of tissue engineering scaffolds because it has adequate biomechanical properties [13], such as biocompatibility and biodegradability, and is naturally decomposed in the body after a certain period [14,15]. However, it is difficult to attach cells on the surface of PCL because it is hydrophobic. To solve this problem, many studies are being conducted to improve the cell adhesion efficiency by modifying the surface of PCL through various treatments, such as NaOH, oxygen plasma, UV irradiation, and gelatin coating [16]. Various studies have attempted to promote cell proliferation by modifying the surface properties of PCL. Cho et al. (2015) produced a hydrophilic PCL nanofiber scaffold by electrospinning a PCL/polyvinylpyrrolidone(PVP)-b-PCL polymer for improving biocompatibility [17], and Ko et al. (2015) analyzed the properties of a plasma-treated electrospun PCL nanofiber scaffold in bone tissue engineering. Jeon et al. (2014) fabricated a surface-modified PCL scaffold with variable nanosized surface roughness using plasma treatment [18].

To improve biological properties, such as the osteogenic differentiation of PCL, we used an oxygen plasma treatment and graphene oxide (GO) coating technology. Many researchers applied the oxygen plasma treatment method because it can etch the surface of PCL in a relatively simple and fast process. In addition, coating GO on the polymer increases the hydrophilicity of the surface. And the surface with increased hydrophilicity promotes cell adhesion. GO has excellent rigidity and biocompatibility [19]; thus, it is used as a transplant platform in stem cell culture [20]. The epoxide, carboxyl, and hydroxyl groups present at the base and edges of GO allows for greater interaction with proteins through covalent, electrostatic, and hydrogen bonding [21]. With this property, GO can reinforce the osteogenic differentiation of stem cells [22]. Lee et al. (2011) studied graphene and GO for enhancing stem cell growth and differentiation [20], and recently, the phase change of GO was evaluated by Yang et al. In addition, there is also active research that GO helps bone regeneration [23,24]. Further, Vera-Sánchez et al. (2016) studied the biocompatibility and potential of a composite coating with graphene oxide and its potential to induce differentiation of human periodontal ligament stem cells [25]. Thus, the enhancement of osteogenic differentiation in GO-based biomaterials was studied [26]. However, in the previous research on 3D printing of GO-based biomaterials, GO was often blended with polymer, which resulted in the excessive consumption of GO. Despite the large consumption of GO, its effect was not significant, since only small amount of GO particles were exposed to the surface. Most of the GO particles were embedded by the PCL rather than exposed on the surface. In this study, a more efficient osteogenic differentiation scaffold model was developed by coating GO on the surface, and the efficiency of the coating was further improved through oxygen plasma treatment.

**Figure 1:**
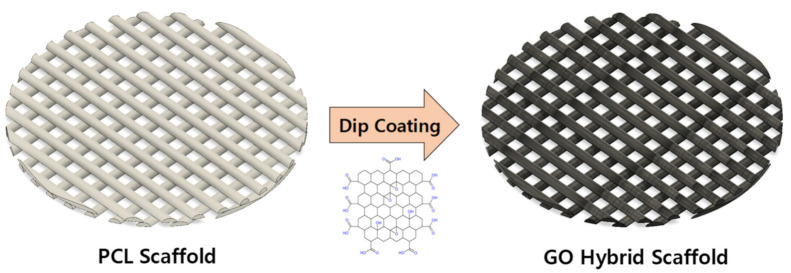
Schematic diagram of scaffold fabrication.

In this study, we aimed to evaluate the enhancement of the osteogenic differentiation capacity of periodontal ligament stem cells by using the synergistic effect of plasma treatment and GO coating on the surface of 3D-printed polycaprolactone scaffolds. First, 3D-printed PCL scaffolds were fabricated, and coating conditions were established according to the oxygen plasma treatment and GO concentration as shown in Figure 1. The physical/chemical properties of the prepared scaffolds were assessed, and the ability of periodontal ligament stem cells to adhere, proliferate, and induce osteogenic differentiation was evaluated.

## 2. Materials and Methods

### 2.1. Fabrication of Plasma-Treated and GO-Coated (P-GO) Scaffold

#### 2.1.1. Scaffolds Fabrication

Scaffolds were fabricated with a material extrusion 3D printer. PCL (MW: 45,000, Polysciences, Warrington, PA, USA) was placed in a stainless-steel syringe, heated to 80 °C using a heating unit, and extruded using a pneumatic pump system. The printed PCL scaffolds were punched out with an 11 mm punch to form a circular scaffold.

#### 2.1.2. Oxygen Plasma Treatment

The plasma treatment was carried out in an oxygen atmosphere in a plasma system (Femto Science, Hwaseong, Korea). Plasma-treated samples were exposed to an oxygen atmosphere for 5 min before the samples were removed from the chamber.

#### 2.1.3. GO Coating

GO was prepared from graphite (Alfa-Aesar, Haverhill, MA, USA) through the modified Hummer’s method [27]. Through dilution in distilled water, the concentration was adjusted to 0.125, 0.250, and 0.5 mg/mL. The plasma-treated scaffolds were coated using a dip-coating method. The GO was washed 3 times with distilled water and sterilized for 30 min with 70% ethanol.

### 2.2. Characterization of the P-GO Scaffold

#### 2.2.1. Raman Spectroscopy

The presence of graphene in the coated scaffolds was confirmed by Raman spectrometry (DXR2xi, Thermofisherscientific, Waltham, MA, USA) at 532 nm [28].

#### 2.2.2. Contact Angle

The hydrophilicity of the scaffolds was confirmed through the water contact angle after the coating with GO. Here, 10 μL of distilled water was dropped on each of the scaffolds to measure the contact angle between the scaffold and water using Easydrop (Kruss, Hamburg, Germany).

#### 2.2.3. Coating Ability and Uniformity

To evaluate the efficiency of the plasma treatment and coating by GO concentration, pictures of the scaffolds were obtained, and the brightness of the pixels was compared through analysis using ImageJ. Five lines were drawn per scaffold at equal intervals with the center of the scaffold farthest from the light source, and the brightness value was calculated as a line. This was averaged to calculate the average values for each plasma coating and GO concentration and mean of the standard deviation was determined.

#### 2.2.4. Field Emission Scanning Electron Microscopy

The morphology of the scaffolds was observed by field emission scanning electron microscopy (FE-SEM) (SUPRA 55VP, Zeiss, Oberkochen, Germany). Scaffolds were sputter coated with 15 nm platinum and observed under the FE-SEM machine at an accelerating voltage of 2 kV.

### 2.3. Cell Viability and Osteoinductivity on the P-GO Scaffold

#### 2.3.1. Cell Culture

Human periodontal ligament stem cells (PDLSCs) were isolated from the dental tissue of adult patients for treatment purposes (Intellectual Biointerface Engineering Center, Dental Research Institute, College of Dentistry, Seoul National University, Institutional IRB approval number: CRI05004). The seeding density on the scaffolds was 5 × 10^4^ cells per scaffold. Incubation was performed at 37.5 °C and 5% CO_2_ in Minimum Essential Medium Eagle-alpha modification (alpha MEM; WELGENE, Gyeongsan, Korea), supplemented with 10% fetal bovine serum (FBS; WELGENE, Gyeongsan, Korea), 1% antibiotics (WELGENE, Gyeongsan, Korea), and 1 mg/mL tyrosine (Sigma-Aldrich, St. Louis, MO, USA) and trypsin-ethylenediaminetetraacetic acid (EDTA) (WELGENE, Gyeongsan, Korea). Osteogenic media was supplied after the cell attachment in the differentiation assay, which was composed of 0.1 μM dexamethasone, 10 μM β–glycerophosphate, and 100 μM ascorbic acid, and added to the proliferation media.

#### 2.3.2. Water-Soluble Tetrazolium Salt-1 (WST-1) Assay

The biological effect of GO was examined using a water-soluble tetrazolium salt-1 (WST-1; Daeillab, Seoul, Korea) assay. The PDLSCs were cultured with culture media comprised of alpha MEM, 10% FBS, 1% antibiotics, and 0.1% tyrosine. GO scaffolds were placed in a 48-well plate and stored at 37 °C for 1 h prior to cell culture. Then, 250 μL of the medium containing PDLSCs was pipetted into each well. Cells were seeded at 5 × 10^4^ cells/well. After 1, 3, and 7 days, the viable cells were determined using a microplate reader (Tecan, Männedorf, Switzerland).

#### 2.3.3. Immunocytochemistry (ICC)

Cells were seeded on the GO scaffolds in 48-well plates at a density of 5 × 10^4^ cells per well and incubated for 1–7 days. The adhered cells were fixed with a 4% paraformaldehyde solution (Sigma-Aldrich, St. Louis, MO, USA) for 60 min, permeabilized with 0.2% Triton X-100 (Sigma-Aldrich, St. Louis, MO, USA) for 15 min, and stained with tetramethylrhodamine(TRITC)-conjugated phalloidin (Millipore, Burlington, MA, USA) and 4,6-diamidino-2-phenylinodole (DAPI; Millipore, Burlington, MA, USA) for 30 min. A fluorescence microscope (Nikon, Tokyo, Japan) was used for acquiring the images of the stained cells.

#### 2.3.4. Alizarin Red S Staining

Cells were seeded on the GO scaffolds in 48-well plates at a density of 1 × 10^6^ cells per well and incubated in osteogenic media for 1 and 2 weeks. After staining the cells with alizarin red S (Sigma-Aldrich, St. Louis, MO, USA), calcium deposition was determined using a microplate reader.

#### 2.3.5. Immunocytochemistry for Assessing Osteogenic Differentiation

Cells were seeded on the GO scaffolds in 48-well plates at a density of 1 × 10^5^ cells per well and incubated in osteogenic media for 2 weeks. The adhered cells were fixed with a 4% paraformaldehyde solution (Sigma-Aldrich, St. Louis, MO, USA) for 60 min, permeabilized with 0.2% Triton X-100 (Sigma-Aldrich, St. Louis, MO, USA) for 15 min, a primary antibody (polyclonal anti-human osteopontin goat IgG, R&D system, Minneapolis, MN, USA) for 1 h, and a secondary antibody (anti-goat IgG fluorescein isothiocyanate (FITC) conjugate) for 1 h, and stained with TRITC-conjugated phalloidin (Millipore, Burlington, MA, USA) and 4,6-diamidino-2-phenylinodole (DAPI; Millipore, Burlington, MA, USA) for 30 min. A fluorescence microscope (Nikon, Tokyo, Japan) was used for acquiring the images of the stained cells.

#### 2.3.6. Alkaline Phosphatase (ALP) Assay

PDLSCs were seeded in a 48-well plate with 1.0 × 10^6^ cells per well in a complete medium; they were treated in an osteogenic differentiation medium. The ALP activity was assessed using the SensoLyte^®^ pNPP Alkaline Phosphatase Assay Kit (Thermo fisher scientific, Waltham, MA, USA) 1 and 4 days after the osteogenic differentiation medium was added, following the manufacturer’s instructions.

### 2.4. Statistical Data Analysis

Statistical analysis was carried out using R Studio for Windows v1.2.5042 (RStudio Inc., Boston, MA, USA). The statistical significance between the control and treatment groups was compared using one-way ANOVA at * *p* < 0.05. The data are reported as the mean ± standard deviation, n = 5.

## 3. Results

### 3.1. Fabrication of the P-GO Scaffold

After fabricating grid-patterned PCL scaffolds using a 3D printer, the scaffolds were exposed to oxygen plasma for 300 s. Then, the scaffolds were coated by immersion in a GO solution of 0.125, 0.25, and 0.5 mg/mL.

### 3.2. Characterization of the P-GO Scaffolds

#### 3.2.1. Physical Properties of the P-GO Scaffolds

**Figure 2:**
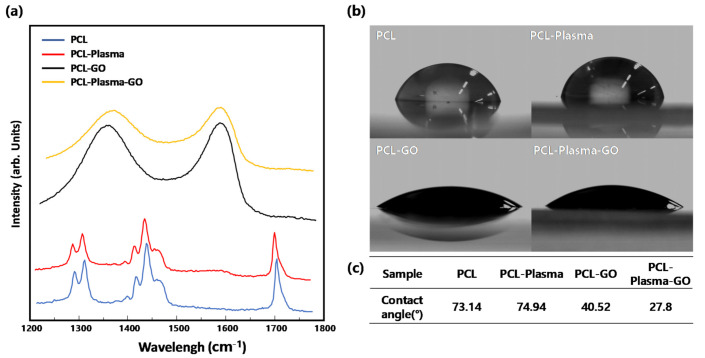
Physical properties of the scaffolds. (**a**) Raman spectra of poly(ε-caprolactone) (PCL), PCL-plasma, PCL-graphene oxide (GO), and PCL-plasma-GO; (**b**) Contact angle and images of PCL, PCL-plasma, PCL-GO, and PCL-plasma-GO; (**c**) Table summarizing the contact angle of PCL, PCL-plasma, PCL-GO, and PCL-plasma-GO.

Raman spectroscopy was used to confirm that the GO produced by the modified Hummer’s method was coated normally. As shown in Figure 2a, unlike the PCL or PCL-plasma treatment group, peaks were observed at 1300 cm^−1^ and 1500 cm^−1^ in the PCL-GO and PCL-plasma-GO treatment groups, respectively. Therefore, GO was coated on the surface of the scaffolds.

To confirm the effect of the hydrophilicity increasing because of the GO coating, 10 µL of water was dropped on each sample, and the water contact angle was measured. Figure 2b shows that, compared to the PCL and PCL-plasma treatment groups, the contact angle of the PCL-GO treatment group decreased by approximately 46%, and that of the PCL-plasma-GO treatment group decreased by approximately 67%. The hydrophilicity of the GO-coated scaffolds was greater than that of the non-coated scaffolds, and the PCL-plasma-GO treatment group was more hydrophilic than the PCL-GO treatment group.

#### 3.2.2. Coating Ability and Uniformity

**Figure 3:**
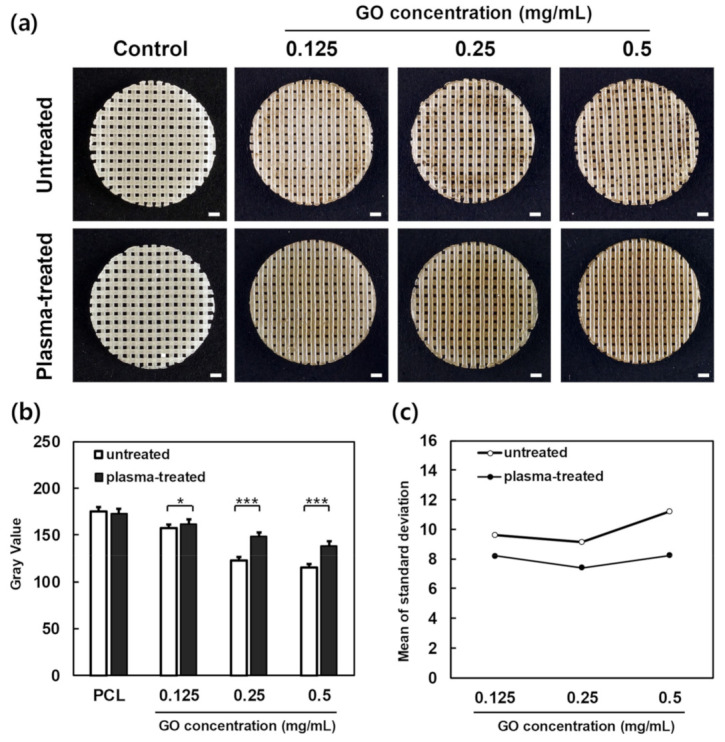
Image analysis of the scaffolds. (**a**) Images of PCL, PCL-GO at 0.125 mg/mL, PCL-GO at 0.25 mg/mL, PCL-GO at 0.5 mg/mL, PCL-plasma, PCL-plasma-GO at 0.125 mg/mL, PCL-plasma-GO at 0.25 mg/mL, and PCL-plasma-GO at 0.5 mg/mL scaffolds. Scale bars; 1.0 mm. (**b**) Pixel intensity by GO density (error bars indicate standard deviations, n = 5). (**c**) Mean of the standard deviation of pixel intensity by GO density (error bars indicate standard deviations, n = 5). * indicates a significant difference (*p* < 0.05), *** indicates a significant difference (*p* < 0.001).

To ensure the uniformity of the scaffold coating, the scaffolds were coated by preparing a GO solution with three concentrations of 0.125, 0.25, and 0.5 mg/m. In Figure 3a it can be seen that the GO-coated scaffolds after plasma treatment performed better than the GO-coated scaffolds without plasma treatment, and the uniformity was greatly enhanced. To confirm this quantitatively, an image analysis method using ImageJ was used. As shown in Figure 3b, the analysis showed that the higher the GO concentration, the lower the gray value. As shown in Figure 3c, the standard deviation of the pixel intensity of the plasma-treated group was approximately 34.5% superior to that of the plasma-untreated group, and the plasma-treated group produced a uniform GO coating compared to the plasma-untreated group.

#### 3.2.3. Morphology of the P-GO Scaffold

**Figure 4:**
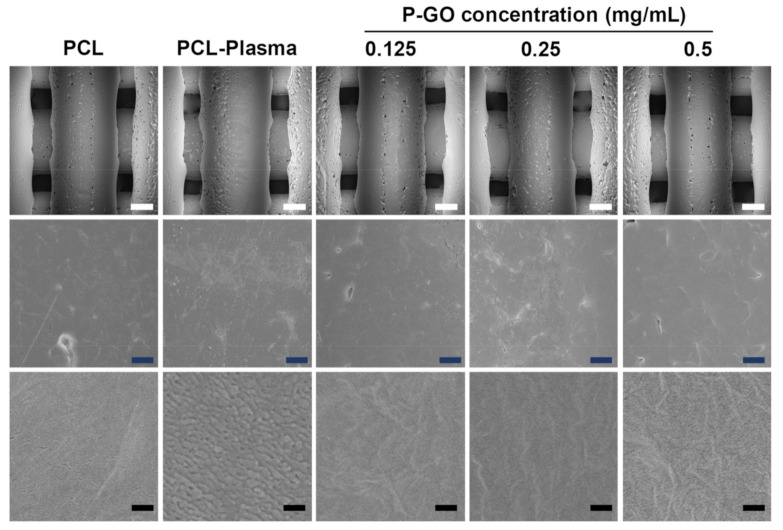
Field emission scanning electron microscopy (FE-SEM) images of scaffolds at 150×, 10,000×, 30,000× magnification. White scale bars; 200 µm, navy scale bars; 10 µm, purple scale bars; 0.25 µm. (P-GO; Oxygen plasma-graphene oxide coating).

The morphology of the scaffolds was determined by FE-SEM. To confirm the microstructure of the scaffolds, the detailed structure was confirmed through FE-SEM. In Figure 4, the surface of the scaffolds is shown. In the 30,000× picture, the scaffold’s surface treated with oxygen plasma for 5 min was etched. For the coating, oxygen plasma was not observed, and GO was assumed to be coated on the etched part.

### 3.3. Cell Viability and Osteoinductivity on the P-GO Scaffolds

#### 3.3.1. Protein Absorption

**Figure 5:**
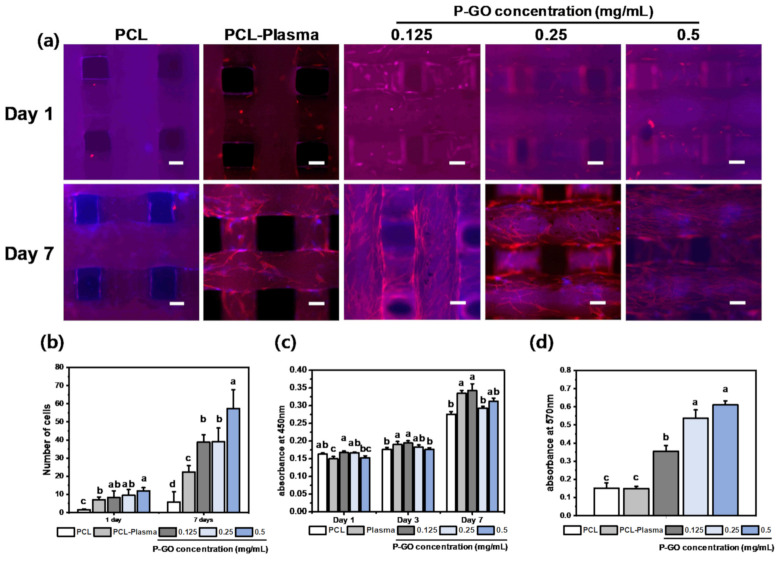
Cell viability on the scaffolds (**a**) Immunocytochemistry images of the scaffolds. Cytoskeleton was stained by tetramethylrhodamine (TRITC)-conjugated phalloidin (red). Cell nuclei were thus visualized by 4,6-diamidino-2-phenylinodole (DAPI, blue). Scale bars; 250 µm. (**b**) Cell counting in immunocytochemistry image (ANOVA, Duncan’s multiple range test, *p* < 0.05). Same letters indicate that there is no significant difference between samples. (**c**) Cell viability results for the periodontal ligament stem cells (PDLSCs) on the PCL, PCL-plasma, and PCL-plasma-GO at 0.125 mg/mL, the PCL-plasma-GO at 0.25 mg/mL, and the PCL-plasma-GO at 0.5 mg/mL. The cell viabilities of the PDLSCs were measured at an absorbance of 450 nm. (**d**) Comparison of bovine serum albumin (BSA) protein absorption of the PCL, PCL-plasma, and with GO scaffolds after incubation for 24 h. The BSA protein absorption was measured at an absorbance of 570 nm (error bars indicate standard deviations, n = 5). (ANOVA, Duncan’s multiple range test, *p* < 0.05). Same letters indicate that there is no significant difference between samples.

From the protein adsorption experiments, there was no difference between the PCL and PCL-plasma scaffolds. Figure 5d shows that the scaffolds coated with 0.125, 0.25, and 0.5 mg/mL GO increased protein adsorption by approximately 238%, 360%, and 409%, respectively, compared to the untreated scaffolds. This confirmed that the graphene coating increased the degree of adsorption between the scaffold and protein.

#### 3.3.2. Immunocytochemistry

To confirm the cell adhesion ability and cytotoxicity of the GO scaffolds, cells were proliferated for 1, 3, and 7 days using PDLSCs, and then, the WST-1 assay was performed. The cytotoxicity of the PDLSCs demonstrated the cytocompatibility of GO. As shown in Figure 5a, there was no significant difference among the samples. The results indicate that the PCL-GO scaffolds are suitable for long-term cell culture. In addition, the ICC image was divided into quarters, and then, the number of cells was counted to evaluate the cell adhesion. As a result of the calculation, the number of cells in the P-GO scaffolds was greater than that of the untreated scaffolds.

Figure 5b shows the number of cells shown in Figure 5a after being counted via ImageJ software. On the 0.5 mg/mL GO-coated experimental group at day 7, a higher number of cells were observed than other experimental groups. Therefore, it was confirmed that the 0.5 mg/mL GO-coated scaffolds were effective in proliferating cells.

#### 3.3.3. Water Soluble Tetrazolium Salt-1 (WST-1) Assay

To confirm the cell adhesion ability and cytotoxicity of the GO scaffolds, cells were proliferated for 1, 3, and 7 days using PDLSCs, and then, the WST-1 assay was performed. The cytotoxicity of the PDLSCs demonstrated the cytocompatibility of GO. Figure 5c shows that there was no significant difference among the samples. The results indicate that the PCL-GO scaffolds are suitable for long-term cell culture.

#### 3.3.4. Alizarin Red S Staining

**Figure 6:**
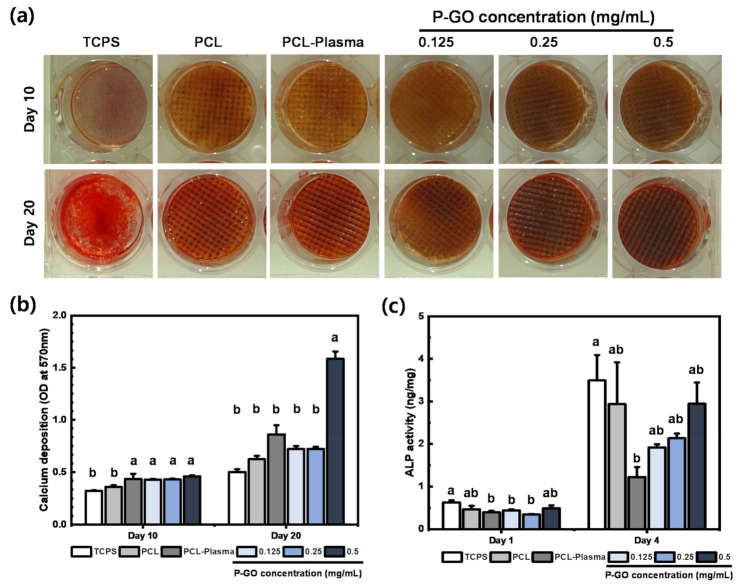
Osteogenic differentiation on the scaffolds (**a**) Calcium deposition visualized by alizarin red S staining after 10 and 20 days of incubation on the scaffolds. (**b**) Quantification demonstrated a significantly higher amount of alizarin red S staining in the PDLSCs differentiated on scaffolds. (**c**) Result of alkaline phosphatase (ALP) assay after 1 day and 4 days. Error bars in Figure 6a,b indicate standard errors. Same letters indicate that there is no significant difference between samples.

To confirm the osteogenic differentiation effect of the P-GO scaffolds, calcium generated during the differentiation process was stained by alizarin red S staining. As shown in Figure 6a,b, the calcium deposition of the P-GO scaffolds increased by 60% compared to the other scaffolds, and the effect of promoting osteogenic differentiation of the 0.5 mg/mL P-GO scaffold was indirectly confirmed.

#### 3.3.5. Alkaline Phosphatase (ALP) Assay

As a result of ALP assay, there was no significant difference in the results between the PCL-plasma scaffolds and the 0.5 mg/mL P-GO scaffolds. However, the absorbance values of P-GO scaffolds showed better ALP activity compared to the PCL-plasma scaffolds. From the results of ALP assay and alizarin red S staining, shown in Figure 6c, it was observed that the initial osteogenic differentiation of the P-GO scaffold was promoted compared to the other experimental groups.

#### 3.3.6. Immunocytochemistry for Assessing Osteogenic Differentiation

**Figure 7:**
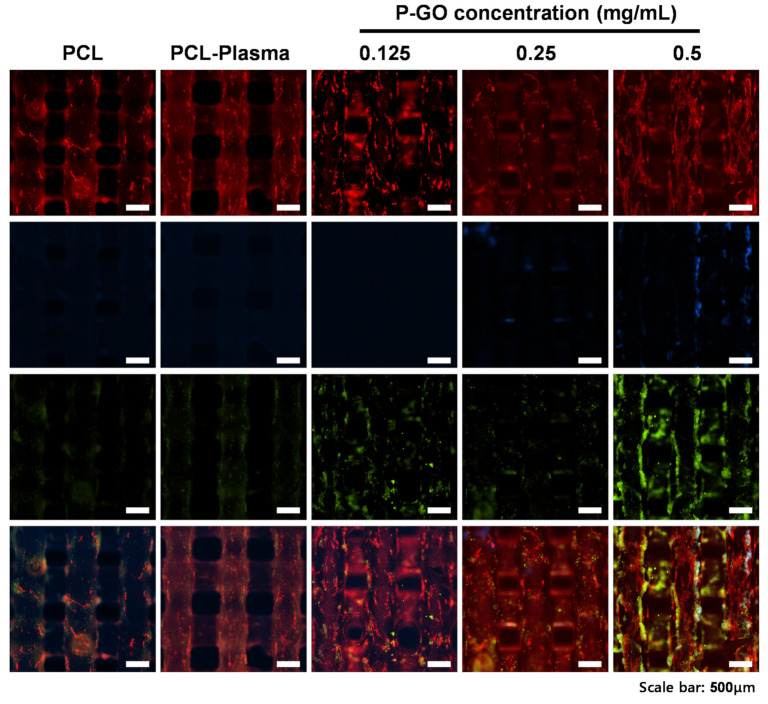
Immunofluorescence images of the scaffolds on day 20. Osteopontin (OPN), actin, and cell nuclei (DAPI) were exhibited as red, green, and blue, respectively. Scale bars; 500 µm.

To confirm the osteogenic differentiation of PDLSCs in the P-GO scaffolds, the presence of osteopontin, an osteogenic differentiation marker, was qualitatively analyzed through ICC. PDLSCs were promoted for osteogenic differentiation for 10 and 20 days using osteogenic media. As a result of observing the images taken through ICC, and shown in Figure 7, it was confirmed that osteopontin, one of the early differentiation markers, was observed more in the P-GO scaffolds. The results indicate that the 0.5 mg/mL P-GO scaffolds are more effective in osteogenic differentiation than the untreated scaffolds.

## 4. Discussion

With the development of 3D printing technology, regenerating living tissues and organs using various biomaterials has been attempted. Among the biomaterials, PCL [29] has been approved by the FDA, has appropriate physical properties, and is widely used because of its biodegradability [30]. However, because of the hydrophobicity of PCL [31], it is difficult to adhere cells to the surface [32], with the result of decreasing cell proliferation [33]. Therefore, reducing the hydrophobicity of PCL and increasing the differentiation ability of stem cells through various post-treatments are also actively being studied [34,35]. The regeneration of periodontal ligament tissues through the above-mentioned technology is also being actively researched. Periodontal ligament tissue is a complex tissue composed of cementum, alveolar bone, periodontal ligament, and gingiva, and once it is damaged, it is difficult to regenerate again [36]. Existing treatments for alveolar bone loss are limited to bone graft filling [37]. To improve this, regenerating complex periodontal ligament tissues by culturing stem cells in scaffolds is required.

In this study, we fabricated P-GO scaffolds by treating the PCL scaffolds with oxygen plasma and then dip-coating GO solution to increase the hydrophilicity, cell proliferation, and bone differentiation ability of the scaffolds. First, GO was synthesized from graphite using the modified Hummer’s method, Raman spectroscopy confirmed that GO was synthesized correctly, and the coating was normally applied on the scaffolds. Peaks were observed at 1350 and 1800 m^−1^, which are the peaks observed in GO. Therefore, the material we synthesized was confirmed to be GO, and GO was normally coated on the scaffolds’ surface. Next, the surface of the coated scaffolds was observed using FE-SEM. In the group treated with only oxygen plasma on PCL at 30,000×, the PCL surface was etched and cut off. However, after oxygen plasma treatment, the surface coated with GO was not observed to be cut off. Thus, GO was coated on the surface cutout after treatment with oxygen plasma so that the surface could not be observed. Next, the change in hydrophobicity for each experimental group was confirmed through the water contact angle, and on the basis of the results of the measurement, the P-GO scaffolds decreased by approximately 62% compared to the untreated group. Thus, the P-GO scaffolds were susceptible to hydrophobicity and increased hydrophilicity compared to the untreated group. Cell adhesion was expected to be facilitated. Here, the coating efficiency of each GO solution was observed using the brightness value through image analysis with ImageJ. From the visual observation before the image analysis, the experimental groups immersed in GO solution without oxygen plasma treatment showed that the coating was not uniformly applied, and the phenomenon was mottled. As the concentration of the GO-coated solution increased, the pixel value decreased on the basis of the quantitative image analysis. When the concentration of the GO solution was higher, the coating appeared darker. In addition, the mean of the standard deviation of the brightness values of the oxygen plasma-treated GO coating group decreased by an average of 20% compared to the standard deviation of the oxygen plasma-treated GO coating group. The oxygen plasma treatment and the coating of GO showed a more even coating compared to the GO coating without treatment, indicating that the GO coating was more efficient.

The purpose of this study was to investigate the proliferation and osteogenic differentiation of stem cells using the synergetic effects of oxygen plasma treatment and GO coating. Before the full-scale cell experiment, bovine serum albumin (BSA) was used to observe the amount of protein adsorbed on each scaffold. From the results of the experiment, the higher the GO concentration, the higher the absorbance at 570 nm. With a higher concentration of GO, the protein adhesion increases and is expected to help cell adhesion. Next, the adhesion and proliferation of PDLSCs were observed using the WST-1 assay. There was no significant difference in the proliferation results on days 1 and 3; however, cell proliferation slightly increased in the scaffolds coated with 0.125 mg/mL of GO on day 7. In addition, the cell proliferation observed through ICC showed that more cytoskeleton and nuclei were present on the GO-coated scaffolds’ surface. Therefore, the GO-coated scaffolds had no cytotoxicity.

Finally, we tried to indirectly confirm the osteogenic differentiation effect through the corresponding scaffolds using alizarin red S staining. By differentiating the cells for 10 and 20 days, calcium deposition increased by 60% in the experimental group coated with the 0.5 mg/mL GO solution. This indirectly confirmed that the osteogenic differentiation capacity of the GO-coated scaffolds was increased.

When the above results are summarized, the coating technique we devised can help bone regeneration by increasing osteogenic differentiation. The new GO coating method using the oxygen plasma treatment improves the differentiation capacity of stem cells, while reducing the amount of GO used. Thus, the regeneration potential of periodontal tissue using stem cells in the scaffolds was confirmed. In addition, the regeneration of bone tissue among periodontal tissues was confirmed. We developed the scaffold for the regeneration of periodontal tissue, which is complex tissue consisting of fibroblasts, cementoblasts, osteoblasts, and undifferentiated mesenchymal stem cells. Several studies investigated the regeneration of periodontal ligament tissue, since it has complex structure and function. Nagata et al. (2017) conducted a study to regenerate periodontal ligaments using mesenchymal stem cells (MSCs) [38], and Mahetab et al. (2020) conducted a study on regenerating gingival fibroblast tissue using a collagen membrane [39]. Zimina et al. (2020) investigated composites containing a biodegradable matrix and bioactive fillers for restoring maxillofacial defects [12]. Our study focused on enhancing the formation of the calcified matrix of PDLSCs, which plays a vital role when the periodontal ligament is combined with the alveolar bone or cementum tissue. Thus, complex periodontal tissue regeneration using PDLSCs could be possible through future research on the regeneration of complex tissues, such as ligaments, gingiva, and bone tissue.

## 5. Conclusions

The objectives of this research were to develop a scaffold with GO coating to improve the bioactivity and osteogenic differentiation ability. Among the experimental groups, the plasma-treated and GO-coated scaffolds showed an improved osteogenic differentiation ability compared to the untreated group or plasma-treated group. In particular, the 0.5 mg/mL P-GO scaffolds showed better osteogenic differentiation ability than the other P-GO scaffolds. The results of this study showed that the P-GO scaffolds improved osteoconductivity. Although we conducted a study on osteogenic differentiation of PDLSCs, which is a basic part of periodontal tissue regeneration, it can be developed as one of the key technologies in advanced complex tissue regeneration if further research is investigated in the future.

## Data Availability

All the experimental data herein presented are available on request from the corresponding author.

## References

[1] Kocialkowski A., Wallace W.A., Prince H. (1990). Clinical experience with a new artificial bone graft: Preliminary results of a prospective study. Injury.

[2] Stravinskas M., Horstmann P., Ferguson J., Hettwer W., Nilsson M., Tarasevicius S., Petersen M.M., McNally M.A., Lidgren L. (2016). Pharmacokinetics of gentamicin eluted from a regenerating bone graft substitute: In vitro and clinical release studies. Bone Jt. Res..

[3] Pinto D., Marques A., Pereira J.F., Palma P.J., Santos J.M. (2020). Long-Term Prognosis of Endodontic Microsurgery—A Systematic Review and Meta-Analysis. Medicina.

[4] Kawaguchi H., Hirachi A., Hasegawa N., Iwata T., Hamaguchi H., Shiba H., Takata T., Kato Y., Kurihara H. (2004). Enhancement of Periodontal Tissue Regeneration by Transplantation of Bone Marrow Mesenchymal Stem Cells. J. Periodontol..

[5] Iwata T., Yamato M., Ishikawa I., Ando T., Okano T. (2014). Tissue Engineering in Periodontal Tissue. Anat. Rec. Adv. Integr. Anat. Evol. Biol..

[6] Pfister A., Landers R., Laib A., Hübner U., Schmelzeisen R., Mülhaupt R. (2004). Biofunctional rapid prototyping for tissue-engineering applications: 3D bioplotting versus 3D printing. J. Polym. Sci. Part A Polym. Chem..

[7] Buchanan C., Gardner L. (2019). Metal 3D printing in construction: A review of methods, research, applications, opportunities and challenges. Eng. Struct..

[8] Chia H.N., Wu B.M. (2015). Recent advances in 3D printing of biomaterials. J. Biol. Eng..

[9] Chen Z., Li Z., Li J., Liu C., Lao C., Fu Y., Liu C., Li Y., Wang P., He Y. (2019). 3D printing of ceramics: A review. J. Eur. Ceram. Soc..

[10] Ramakrishna S., Mayer J., Wintermantel E., Leong K.W. (2001). Biomedical applications of polymer-composite materials: A review. Compos. Sci. Technol..

[11] Guvendiren M., Molde J., Soares R.M., Kohn J. (2016). Designing Biomaterials for 3D Printing. ACS Biomater. Sci. Eng..

[12] Zimina A., Senatov F., Choudhary R., Kolesnikov E., Anisimova N., Kiselevskiy M., Orlova P., Strukova N., Generalova M., Manskikh V. (2020). Biocompatibility and Physico-Chemical Properties of Highly Porous PLA/HA Scaffolds for Bone Reconstruction. Polymer.

[13] Baptista P.M., Orlando G., Mirmalek-Sani S.-H., Siddiqui M., Atala A., Soker S. (2009). Whole organ decellularization—A tool for bioscaffold fabrication and organ bioengineering. Proceedings of the 2009 Annual International Conference of the IEEE Engineering in Medicine and Biology Society.

[14] Niinomi M. (2008). Metallic biomaterials. J. Artif. Organs.

[15] Park J.B., Kim Y.K. (2003). Metallic Biomaterials.

[16] Sáenz A., Rivera E., Brostow W., Castaño V.M. (1999). Ceramic biomaterials: An introductory overview. J. Mater. Educ..

[17] Khorasani A.M., Gibson I., Goldberg M., Nomani J., Littlefair G. (2016). Machinability of Metallic and Ceramic Biomaterials: A Review. Sci. Adv. Mater..

[18] Park J.B. (2012). Biomaterials Science and Engineering.

[19] Zhang X., Yin J., Peng C., Hu W., Zhu Z., Li W., Fan C., Huang Q. (2011). Distribution and biocompatibility studies of graphene oxide in mice after intravenous administration. Carbon.

[20] Wang K., Ruan J., Song H., Zhang J., Wo Y., Guo S., Cui D. (2010). Biocompatibility of Graphene Oxide. Nanoscale Res. Lett..

[21] Yildirim E.D., Besunder R., Pappas D., Allen F., Güçeri S., Sun W. (2010). Accelerated differentiation of osteoblast cells on polycaprolactone scaffolds driven by a combined effect of protein coating and plasma modification. Biofabrication.

[22] Fu C., Yang X., Tan S., Song L. (2017). Enhancing Cell Proliferation and Osteogenic Differentiation of MC3T3-E1 Pre-osteoblasts by BMP-2 Delivery in Graphene Oxide-Incorporated PLGA/HA Biodegradable Microcarriers. Sci. Rep..

[23] Holt B.D., Wright Z.M., Arnold A.M., Sydlik S.A. (2017). Graphene oxide as a scaffold for bone regeneration. Wiley Interdiscip. Rev. Nanomed. Nanobiotechnol..

[24] Lu J., He Y.-S., Cheng C., Wang Y., Qiu L., Li D., Zou D. (2013). Self-Supporting Graphene Hydrogel Film as an Experimental Platform to Evaluate the Potential of Graphene for Bone Regeneration. Adv. Funct. Mater..

[25] Vera-Sánchez M., Aznar-Cervantes S., Jover E., García-Bernal D., Oñate-Sánchez R.E., Hernández-Romero D., Moraleda J.M., Collado-González M., Rodríguez-Lozano F.J., Cenis J.L. (2016). Silk-Fibroin and Graphene Oxide Composites Promote Human Periodontal Ligament Stem Cell Spontaneous Differentiation into Osteo/Cementoblast-Like Cells. Stem Cells Dev..

[26] Lee W.C., Lim C.H.Y.X., Shi H., Tang L.A.L., Wang Y., Lim C.T., Loh K.P. (2011). Origin of Enhanced Stem Cell Growth and Differentiation on Graphene and Graphene Oxide. ACS Nano.

[27] Zaaba N., Foo K., Hashim U., Tan S., Liu W.-W., Voon C. (2017). Synthesis of Graphene Oxide using Modified Hummers Method: Solvent Influence. Procedia Eng..

[28] Díez-Betriu X., Álvarez-García S., Botas C., Álvarez P., Sánchez-Marcos J., Prieto C.A., Menéndez R., De Andrés A. (2013). Raman spectroscopy for the study of reduction mechanisms and optimization of conductivity in graphene oxide thin films. J. Mater. Chem. C.

[29] Serrano M.C., Pagani R., Vallet-Regı M., Pena J., Ramila A., Izquierdo I., Portolés M.T. (2004). In vitro biocompatibility assessment of poly (ε-caprolactone) films using L929 mouse fibroblasts. Biomaterials.

[30] Abedalwafa M., Wang F., Wang L., Li C. (2013). Biodegradable poly-epsilon-caprolactone (PCL) for tissue engineering applications: A review. Rev. Adv. Mater. Sci..

[31] Xu T., Liang Z., Ding B., Feng Q., Fong H. (2018). Polymer blend nanofibers containing polycaprolactone as biocompatible and biodegradable binding agent to fabricate electrospun three-dimensional scaffolds/structures. Polymer.

[32] Jacobs T., De Geyter N., Morent R., Desmet T., Dubruel P., Leys C. (2011). Plasma treatment of polycaprolactone at medium pressure. Surf. Coat. Technol..

[33] Suntornnond R., An J., Chua C.K. (2016). Effect of gas plasma on polycaprolactone (PCL) membrane wettability and collagen type I immobilized for enhancing cell proliferation. Mater. Lett..

[34] Kim K., Lee K., Cho K., Park C. (2002). Surface modification of polysulfone ultrafiltration membrane by oxygen plasma treatment. J. Membr. Sci..

[35] Luo Y., Shen H., Fang Y., Cao Y., Huang J., Zhang M., Dai J., Shi X., Zhang Z. (2015). Enhanced Proliferation and Osteogenic Differentiation of Mesenchymal Stem Cells on Graphene Oxide-Incorporated Electrospun Poly(lactic-co-glycolic acid) Nanofibrous Mats. ACS Appl. Mater. Interfaces.

[36] Duan X., Tu Q., Zhang J., Ye J., Sommer C., Mostoslavsky G., Kaplan D., Yang P., Chen J. (2011). Application of induced pluripotent stem (iPS) cells in periodontal tissue regeneration. J. Cell. Physiol..

[37] Tamimi F., Torres J., Bassett D., Barralet J., Cabarcos E.L. (2010). Resorption of monetite granules in alveolar bone defects in human patients. Biomaterials.

[38] Nagata M., Iwasaki K., Akazawa K., Komaki M., Yokoyama N., Izumi Y., Morita I. (2017). Conditioned Medium from Periodontal Ligament Stem Cells Enhances Periodontal Regeneration. Tissue Eng. Part A.

[39] Abdal-Wahab M., Ghaffar K.A.A., Ezzatt O.M., Hassan A.A.A., El Ansary M.M.S., Gamal A.Y. (2020). Regenerative potential of cultured gingival fibroblasts in treatment of periodontal intrabony defects (randomized clinical and biochemical trial). J. Periodontal Res..

